# Remote ischemic preconditioning improves ileal microvascular oxygenation during rodent hemorrhagic shock without improving variables of microcirculation and mitochondrial respiration

**DOI:** 10.1038/s41598-025-29245-2

**Published:** 2025-12-04

**Authors:** Stefan Hof, Leandra Krüll, Liana Stepanyan, David Jeikowski, Carsten Marcus, Anne Kuebart, Borna Relja, Anna Herminghaus, Christian Vollmer, Inge Bauer, Olaf Picker, Richard Truse

**Affiliations:** 1 Department of Anesthesiology, University Hospital Duesseldorf, Duesseldorf, Germany; 2 Department of Anesthesia, Critical Care and Pain Medicine, Beth Israel Deaconess Medical Center, Harvard Medical School, Boston, MA USA; 3 Department of Trauma, Hand, Plastic and Reconstructive Surgery, Translational and Experimental Trauma Research, University Hospital Ulm, Ulm, Germany

**Keywords:** RIPC, Tissue protection, Microvascular blood flow, Bleeding, Trauma, Animal model, Intestine, Trauma, Experimental models of disease

## Abstract

Hemorrhagic shock is characterized by decreased microvascular blood flow, leading to a critical depletion of tissue oxygenation, particularly within the gastrointestinal tract. The integrity of the intestinal barrier, however, plays a crucial role in maintaining systemic homeostasis. Remote ischemic preconditioning (RIPC), achieved through transient, repeated occlusion of blood flow to a limb, has been shown to exert beneficial effects on various tissues under hypoxic conditions. These effects are at least in part attributed to circulating humoral mediators with vasoactive properties. This study aimed to investigate the effect of RIPC - implemented via intermittent bilateral hind limb perfusion occlusion - on intestinal microvascular oxygenation, regional microcirculation, and mitochondrial function in a fixed-pressure model of hemorrhagic shock in rats with subsequent autologous blood transfusion. RIPC ameliorated the decrease of microvascular oxygenation in the ileum (shock: 34 ± 4% vs. shock + RIPC: 48 ± 4%), but not in the colon (shock: 51 ± 2% vs. shock + RIPC: 50 ± 4%). RIPC had neither an effect on regional microcirculation nor on mitochondrial respiration assessed ex vivo post hemorrhage and transfusion. These findings suggest that RIPC may improve intestinal tissue oxygenation in hemorrhagic shock independently of regional microcirculation and mitochondrial respiration and that this protective effect of RIPC might vary between different sections of the gastrointestinal tract. The mechanism by which RIPC improves ileal tissue oxygenation has to be clarified in future studies.

**Supplementary Information:**

The online version contains supplementary material available at 10.1038/s41598-025-29245-2.

## Introduction

Hemorrhagic shock is characterized by reduced microvascular blood flow with subsequent depletion of the cellular oxygen reserve^1^. The transfusion of blood products was reported to improve microvascular blood flow and reduce regional hypoxia^2^. Therefore, blood transfusion might be suitable to protect tissues from progredient tissue injury and cell damage. However, blood products are a limited resource that is not universally available and liberal transfusion regimes have been reported to be associated with increased morbidity and mortality^3^. The latter could be the result of reperfusion injury. Taken together, alternative strategies without systemic side effects are needed to compliment shock resuscitation and improve tissue oxygenation in patients suffering from hemorrhagic shock. Regional microcirculation and mitochondrial respiration are major determinants of tissue oxygenation and were defined as *the critical unit of shock*^4^. Therapeutic interventions that aim to improve one of these critical compartments of final oxygen delivery and energy metabolism could therefore improve tissue oxygenation, reduce regional hypoxia and induce tissue protection under conditions of restricted oxygen delivery.

Investigating the gastrointestinal tract during hemorrhagic shock and subsequent blood transfusion could be of outstanding importance due to two reasons. First, adverse effects of hemorrhagic shock on intestinal microcirculation and mitochondrial function are pronounced due to accentuated adrenergic vasoconstriction^5^. Monitoring gastrointestinal microcirculation and mitochondrial function could therefore be helpful to detect early shock states with in part maintained hemodynamic variables and to guide resuscitation. Second, intestinal hypoxia and local tissue injury can initiate intestinal barrier shutdown leading to bacterial translocation and increased exposure to pathogen-associated components^6,7^. Protecting gastrointestinal tissue by improving regional tissue oxygenation could therefore reduce late onset inflammation after acute hemorrhage and reduce overall mortality.

Murry et al. described an endogenous protective mechanism called ischemic preconditioning (IPC) in a model of canine myocardial infarction^8^. They reported that short, non-lethal cycles of ischemia and reperfusion can protect the conditioned tissue from a subsequent hypoxic challenge. Surprisingly, not only the preconditioned tissue, but also tissues that depend on other vascular units were protected by IPC making this concept suitable for translation into the clinical application. To underline the opportunity of systemic tissue protection, this phenomenon was called remote ischemic preconditioning (RIPC)^9^. To date, the transduction and the mechanisms of RIPC-mediated tissue protection have not been fully elucidated. Since some of the effects attributed to RIPC have been reported to be related to humoral factors with microvascular activity^10^, we previously investigated the effect of RIPC as a potential strategy to protect intestinal integrity on ileal and colonic microvascular oxygenation, intestinal microcirculation and mitochondrial respiration in a model of hemorrhagic shock in rats^11^.

Contrary to publications that reported beneficial effects of IPC and RIPC on intestinal tissue integrity^12–15^, RIPC did not reduce histological and functional tissue injury in this study. Further, neither microvascular oxygenation as an early indicator of regional hypoxia nor microvascular blood flow and mitochondrial respiration as determinants of terminal oxygen supply and energy metabolism were altered by RIPC. It has to be mentioned, that these experiments were performed in a model of isolated hemorrhagic shock without hemodynamic resuscitation, whereas the overall tissue damage seems to be attributed at least in part to reperfusion injury^16,17^ in models of complete vascular occlusion. Conclusively, the beneficial effects of RIPC on tissue integrity might be linked to an increased resistance of the investigated tissue against reperfusion injury.

To provide a more detailed insight into RIPC derived tissue protection, we conceptualized this study to investigate the effect of RIPC on microvascular oxygenation, regional microcirculation and mitochondrial respiration in a combined model of hemorrhagic shock with subsequent shed blood transfusion. We hypothesized, that RIPC exerts beneficial effects on intestinal tissue integrity and microvascular oxygenation by enhancing microvascular blood flow and mitochondrial respiration during early hemodynamic resuscitation.

## Methods

**Fig. 1:**
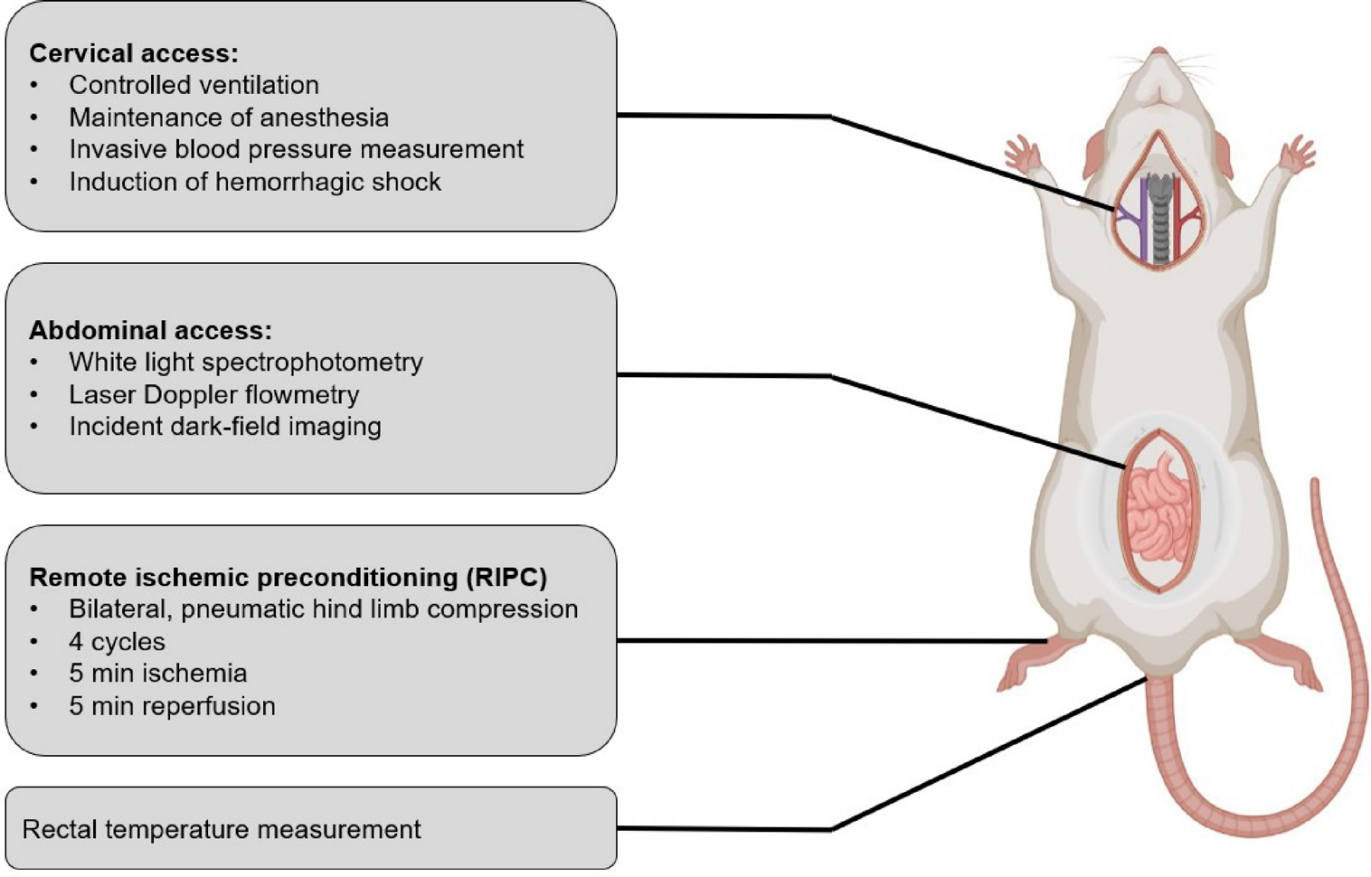
Instrumentation and experimental preparation. This figure has been adapted from 55. Some components of this figure were created with BioRender.

All methods were carried out in accordance with relevant guidelines and regulations. The experimental protocol and the findings of the recent study are reported in accordance with the ARRIVE guidelines. All experiments were approved by the local authorities for experimental animal care and use (Landesamt für Natur, Umwelt und Verbraucherschutz, Recklinghausen, Germany, AZ. 81-02.04.2018 A308). 48 healthy, male Wistar rats (350 ± 35 g body weight) were bred for experimental purpose and derived from the animal research facility of the Heinrich-Heine-University Duesseldorf (ZETT, Zentrale Einrichtung für Tierforschung und wissenschaftliche Tierschutzaufgaben, Duesseldorf, Germany). Animals were anesthetized (sodium pentobarbital 100 mg·kg^− 1^, i.p.), placed in supine position on a heating plate and received standardized instrumentation (Fig. 1). The experimental setting included tracheostomy for mechanical, volume controlled ventilation (V_T_: 6 ml·kg^− 1^; PEEP: 2 cmH_2_O; I:E: 1:2), an invasive arterial blood pressure measurement, and central venous cannulation via a cervical surgical access. Further, median laparotomy was performed to enable intestinal microvascular assessment. After the establishment of a central venous catheter, anesthesia was continued via a continuous infusion (sodium pentobarbital 10 mg·kg^− 1^·h^− 1^, i.v.). Animals received fluids with a fixed rate of 8 ml·kg^− 1^·h^− 1^ to prevent thrombus formation at the catheter and to partially compensate fluid loss via the abdominal cavity. Since hypercapnia^18^ and hypothermia^19^ exerted beneficial effects on microvascular oxygenation in hemorrhagic dogs, we verified normocapnic ventilation via intermittent blood gas analysis and normothermia by continuous rectal temperature measurement. Steady-state conditions were defined as the stability of macro- and microcirculatory variables, registered normothermia and physiological ventilation variables for at least 10 min.

**Fig. 2:**
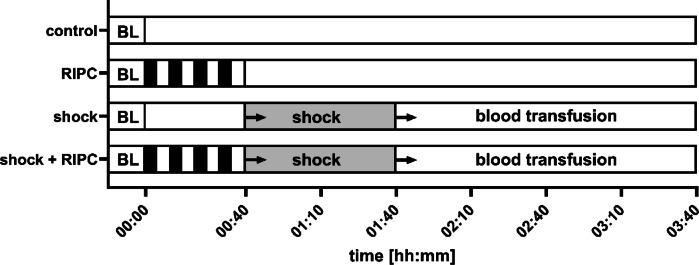
Experimental protocol. After the registration of baseline conditions (BL), animals received either remote ischemic preconditioning (RIPC), hemorrhagic shock (shock) or a combination of both treatments (shock + RIPC). In experimental groups with hemorrhagic shock shed blood was transfused after 1 h of shock and animals were observed for 2 h. Animals without RIPC and hemorrhagic shock with subsequent shed blood transfusion served as control (control).

Baseline measurements were performed at the beginning of the experiments after a stabilization period of 15 min and the animals were randomized to one of four experimental groups with 12 individuals each (Fig. 2). In experimental groups with remote ischemic preconditioning (RIPC) pneumatic cuffs were placed around both hindlimbs and were insufflated simultaneously to 200 mmHg for 5 min. In combination with a deflation of the hindlimb cuffs followed by 5 min of reperfusion one preconditioning cycle was completed. Animals received 4 cycles of bilateral hindlimb ischemic preconditioning. In animals without RIPC, cuffs were placed around both hindlimbs without intermittent insufflation. Subsequently, a fixed-pressure hemorrhagic shock was induced by acute blood withdrawal via the arterial catheter. Systemic blood pressure driven bleeding was supported by moderate, manual blood aspiration to reach the targeted mean arterial blood pressure of 40 ± 5 mmHg within a period of 5 min. Fixed-pressure hemorrhage was then held within the predetermined systemic blood pressure limits for one hour. In case of endogenous hemodynamic stabilization e.g. by fluid mobilization from the extravascular compartment or endogenous catecholamine release, further blood samples were removed to reestablish the targeted blood pressure level. The shed blood was stored with citrate-phosphate-dextrose (Santa Cruz biotechnology Inc., Dallas USA), and filtered (Sangofix, pore size: 200 μm, B. Braun Melsungen, Germany) to retract residual blood clots. Shed blood was transfused after one hour of hemorrhagic shock induction followed by a 2 h observational period. Shed blood was transfused without a fixed time protocol considering systemic circulatory variables to avoid iatrogenic volume overload. Body core temperature was held within the physiological range for rats by external warming. Animals that were randomized to the normovolemic experimental groups did not receive hemorrhage-transfusion and were observed for a total of 3 h. At the end of the experiments, animals were euthanized in deep anesthesia (additional injection of 15 mg·kg^− 1^, i.v. sodium pentobarbital) by exsanguination. Tissue and plasma samples were collected for further analysis.

### Microvascular oxygenation and microcirculation

#### White light spectrophotometry and laser doppler flowmetry

White light consisting of wavelengths between 450 and 1000 nm was used to illuminate intestinal tissue and measure microvascular oxygenation (µHbO_2_) in-vivo. Simultaneously, laser Doppler flowmetry was used to determine microvascular blood flow (µflow). As described in previous studies^11,20^, light is wavelength-dependently absorbed by erythrocytes^21^ and scattered by cellular and interstitial matrix components^22^. In consequence, the reflected light signal differs from the emitted light in terms of intensity and spectral wavelength distribution. The more hemoglobin molecules are contained in a predetermined tissue sample, the larger is the proportion of absorbed light and the lower the intensity of the reflected light. At the same time, the spectrum of the reflected light depends on the averaged hemoglobin oxygen saturation. Therefore, microvascular oxygenation (µHBO_2_; [%]) can be determined from the reflected light signal, if the reflected light is detected at the organs surface. Averaged µHbO_2_ mainly indicates postcapillary oxygen saturation as a measure of regional hypoxia and local oxygen reserve, since the largest amount of microvascular blood volume is stored in the venous compartment of the microcirculation. In addition, laser light with 820 nm wavelength was used to determine microvascular blood flow (µflow; [aU]) based on the principle of laser Doppler flowmetry^23^. Moving particles such as erythrocytes induce a frequency shift, depending on the flow velocity. The reflected laser light can be used to calculated the relative frequency shift, that is closely related to microvascular blood flow velocity.

Both principles, white light spectrophotometry and laser Doppler flowmetry, were performed simultaneously by one device to evaluate microvascular oxygenation and microvascular blood flow of the same gastrointestinal section. This device (O2C LW 2222, LEA Medizintechnik GmbH, Gießen, Germany) is able to determine microvascular variables of two different sites using two independent measuring probes (Flat Probe LFX-2, LEA Medizintechnik GmbH, Gießen, Germany). We aimed to assess two sections of the gastrointestinal tract to investigate, if the effect of RIPC depends on the region of microcirculatory and mitochondrial assessment. We chose the terminal ileum and the ascending colon for microvascular assessment since both regions can be identified easily by prior externalization of the ileocecal junction region. Further, the colon is exposed to a plurality of pathogens due to the extensive load of bacteria in the colonic lumen. Despite the close vicinity between ileum and colon, both compartments of the gastrointestinal tract belong to different vascular beds with an independent vascular blood supply. The photometric signals were analyzed by an investigator blinded to the experimental protocol.

#### Incident dark-field imaging

The microcirculation of the ascending colon was examined using incident dark field (IDF) imaging (CytoCam, Braedius Medical, Huizen, The Netherlands)^24^. Since ileal loops appear to have a smaller diameter, an additional measurement to evaluate the ileal microcirculation using IDF-imaging was not performed due to technical reasons. In detail, the IDF-probe was placed on the serosal surface of the colon and tissue was illuminated with pulsed green light of 530 nm wavelength. In contrast to other experimental studies investigating the intestinal microcirculation during hemorrhagic shock in large animal models^2,25,26^, the intestine was not opened to avoid regional tissue injury by surgical preparation that could lead to microcirculatory alterations. The emitted light is reflected by interstitial cells and matrix components and can be detected by the same device at the organs surface. Since both, oxygenated and deoxygenated hemoglobin, lead to complete extinction of green light, erythrocytes appear as dark light recesses^27^. Five video sequences each were recorded under baseline conditions (00:00), after RIPC-treatment (00:40), in the end of hemorrhagic shock (01:40) and two times after shed blood transfusion (02:40; 03:40). After stabilization of the video sequences the total vessel density (TVD) was calculated for small vessels by an automated software that is provided by the manufacturer (CytoCamTools Analysis software Version 2, Braedius Medical, Huizen, The Netherlands). Further, a blinded investigator scored the stabilized video sequences as reported previously to access microvascular flow index (MFI) and heterogeneity index (HGI) of small vessels^28^. The scoring was supervised by a team of researchers with over five years of experience each in IDF imaging and microvascular assessment. Only videos that met internationally published quality standards were analyzed and evaluated^29,30^.

### Mitochondrial respiration and oxidative stress

#### Respirometry

Ileal and colonic tissue samples were harvested at the end of the experiments to evaluate mitochondrial respiration as described previously in tissue homogenates^31^. No additional experiments have been performed to assess mitochondrial respiration after isolated hemorrhagic shock, since this was subject to a preliminary study^11^. After enzymatic homogenization, respirometry was performed using a Clark-type electrode (model 782, Strathkelvin instruments, Glasgow, Scotland). Substrates for complex I (glutamate, 2.5 mM; Fluka Chemie GmbH Buchs, Switzerland and malate, 2.5 mM; Serva Electrophoresis GmbH, Heidelberg, Germany) and complex II (succinate, 5 mM; Sigma-Aldrich Corporation, St. Louis, MO, USA) of the respiratory chain were provided separately and oxygen consumption was measured under baseline conditions (state 2) and after stimulation by adenosine diphosphate (Sigma-Aldrich Corporation, St. Louis, MO, USA, 125 µM) substitution (state 3). The measurement was terminated after complete metabolization of adenosine diphosphate (state 4). Respiration rates are expressed as nanomole oxygen consumption per minute per milligram protein (nmol·min^− 1^·mg^− 1^). Protein concentration was determined by the Lowry method^32^. The respiratory control index (RCI = state 3 / state 2; RCI* = state 3 / state 4) and the ADP/O-ratio (ADP/O-ratio = ADP added / O_2_ consumption in state 3) were calculated as integrative indicators of mitochondrial coupling between the respiratory chain and the oxidative phosphorylation, and the respiratory efficiency at maximal stimulation, respectively. The measurements were carried out by an investigator blinded to the experimental protocol.

#### Tissue malondialdehyde concentration

The amount of tissue malondialdehyde (MDA) as a degradation product of lipid peroxidation and oxidative stress was measured ex-vivo^33^. Tissue samples of ileum and colon were homogenized and mixed with 0.6% thiobarbituric acid. MDA and thiobarbituric acid molecules form a metabolite when heated to 95 °C for 45 min that can be detected spectrophotometrically at 535 nm and 520 nm^34^. MDA concentration was normalized to tissue protein concentration as determined by the Lowry method^32^ (MDA [nmol] · protein [mg^− 1^]). The measurements were carried out by an investigator blinded to the experimental protocol.

### Intestinal tissue injury

Intestinal tissue injury was determined by histological scoring according to morphological descriptions from Chiu et al.^35^ and determination of the plasmatic D-lactate concentration. For this purpose, ileal tissue samples from about 1 cm length were harvested after the experiments, fixed with formaldehyde and embedded in paraffine blocks. Slides of 8 μm thickness were prepared for subsequent HE-staining. Then, gradual loss of histological adherence between the epithelial cell layer and the stromal grounding was assessed by a blinded investigator. Plasmatic D-lactate concentration was determined using a colorimetric MTT-formazan based assay (D-lactate, colorimetric assay kit, MAK058-1KT, Sigma-Aldrich, Germany).

### Statistics

A required group size of *n* = 12 animals is needed to achieve a power > 0.8 for the detection of differences between the experimental groups at a given α < 0.05 and η^2^ of 0.5 (G*Power Version 3.1.9.2).

Micro- and microcirculatory variables were obtained under steady-state conditions during the last 5 min of each experimental period. Normal distribution was tested using QQ-plots. Analysis of variance in combination with a Bonferroni post-hoc test (macro- and microcirculatory variables) or Šidák correction for multiple comparison between the experimental groups (plasmatic D-lactate concentration) were used to analyze normally distributed variables (GraphPad Prism version 6.05 for Windows, GraphPad Software, La Jolla California United States). Data are presented as mean ± standard error of the mean for *n* = 12. Variables of mitochondrial respiration, lipid peroxidation and histological tissue injury did not follow normal distribution or were classified as categorial variables and therefore were analyzed using Kruskal-Wallis testing and Dunn´s multiple comparisons. Values are reported as median ± interquartile range.

## Results

### Mean arterial blood pressure

**Fig. 3:**
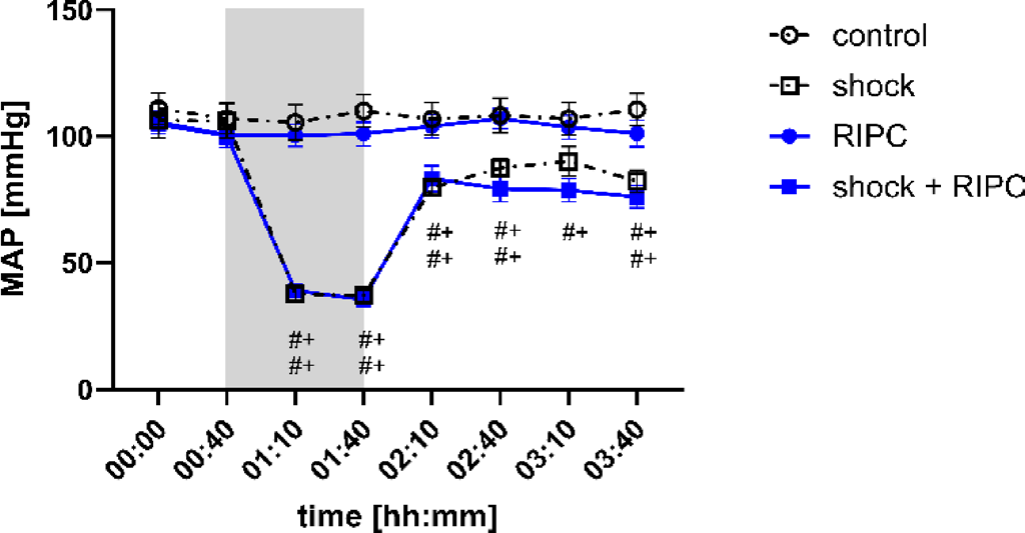
Mean arterial pressure. Time-related changes of the mean arterial blood pressure (MAP; [mmHg]) after control treatment, remote ischemic preconditioning (RIPC) and/or hemorrhagic shock with subsequent shed blood transfusion (shock). 1 h of hemorrhagic shock is marked grey. Data are presented as mean ± SEM for *n* = 12 Wistar-rats *p* ≤ 0.05. #: vs. individual baseline; +: control vs. shock or RIPC vs. shock + RIPC.

MAP remained stable over time in normovolemic animals. According to the experimental protocol of fixed-pressure hemorrhage, MAP decreased after the induction of hemorrhagic shock and reached MAP values of 40 ± 5 mmHg which were kept for one hour (Fig. 3). These changes were significant compared to the individual baseline values and the normovolemic control group. Shed blood transfusion increased MAP in all animals with hemorrhagic shock but failed to reestablish baseline values two hours after hemorrhagic shock. RIPC had no effect on MAP under physiological conditions, during hemorrhagic shock, and after shed blood transfusion.

### Arterial blood gas analysis

**Fig. 4:**
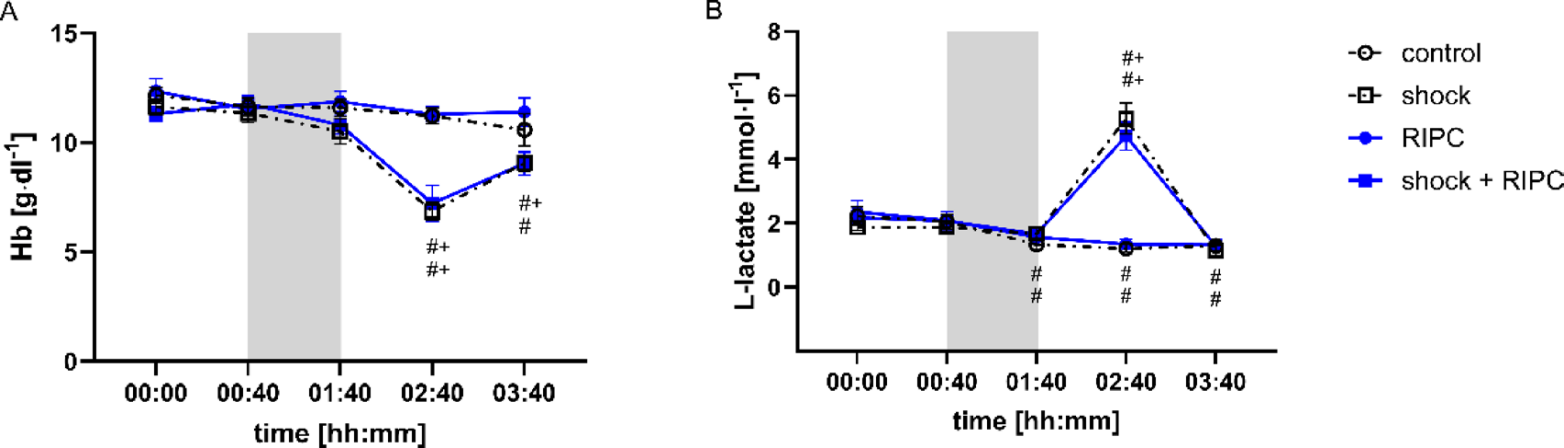
Arterial hemoglobin and L-lactate concentration. Time-related changes of arterial hemoglobin (Hb; [g·dl^− 1^]) and L-lactate concentration [mmol·l^− 1^] after control treatment, remote ischemic preconditioning (RIPC) and/or hemorrhagic shock with subsequent shed blood transfusion (shock). 1 h of hemorrhagic shock is marked grey. Data are presented as mean ± SEM for *n* = 12 Wistar-rats *p* ≤ 0.05. #: vs. individual baseline; +: control vs. shock or RIPC vs. shock + RIPC.

There was no difference of blood hemoglobin concentration between the experimental groups under baseline conditions (Fig. 4A). One hour after the transfusion of shed blood arterial hemoglobin concentration decreased in both shock groups and remained lower than baseline values until the end of the experiment. Concordant to decreased blood hemoglobin concentration arterial L-lactate concentration significantly increased after shed blood transfusion (Fig. 4B). Two hours after blood transfusion there was no difference of arterial L-lactate concentration between normovolemic and hemorrhagic experimental groups. All results were independent of prior RIPC-treatment. RIPC treatment had no effect on acid-base status or arterial blood gases under either physiological or hemorrhagic circulatory conditions (Fig. S1).

### White light spectrophotometry

**Fig. 5:**
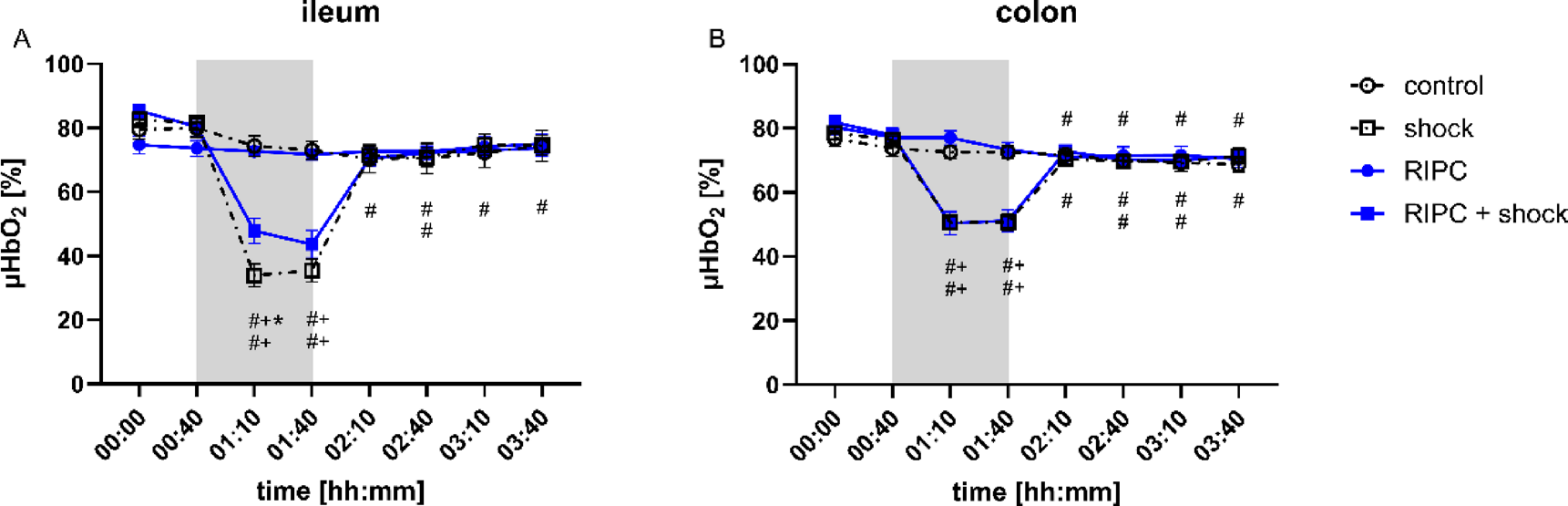
White light spectrophotometry. Time-related changes of ileal (**A**) and colonic (**B**) microvascular oxygenation (µHbO_2_; [%]) after control treatment, remote ischemic preconditioning (RIPC) and/or hemorrhagic shock with subsequent shed blood transfusion (shock). 1 h of hemorrhagic shock is marked grey. Data are presented as mean ± SEM for *n* = 12 Wistar-rats, *p* ≤ 0.05. *: control vs. RIPC or shock vs. shock + RIPC; #: vs. individual baseline; +: control vs. shock or RIPC vs. shock + RIPC.

At baseline, there was no significant difference of ileal and colonic µHbO_2_ between the experimental groups (Fig. 5). µHbO_2_ decreased after the induction of hemorrhagic shock in both sections of the gastrointestinal tract, whereas subsequent shed blood transfusion reestablished baseline values in the control group. Prior RIPC-treatment did not exert a beneficial effect on intestinal µHbO_2_ under physiological conditions. In animals with hemorrhagic shock RIPC improved µHbO_2_ of the ileum during the first 30 min of acute hemorrhage without a beneficial effect on colonic µHbO_2_. RIPC had no effect on ileal and colonic µHbO_2_ two hours after the transfusion of shed blood.

### Laser doppler flowmetry

**Fig. 6:**
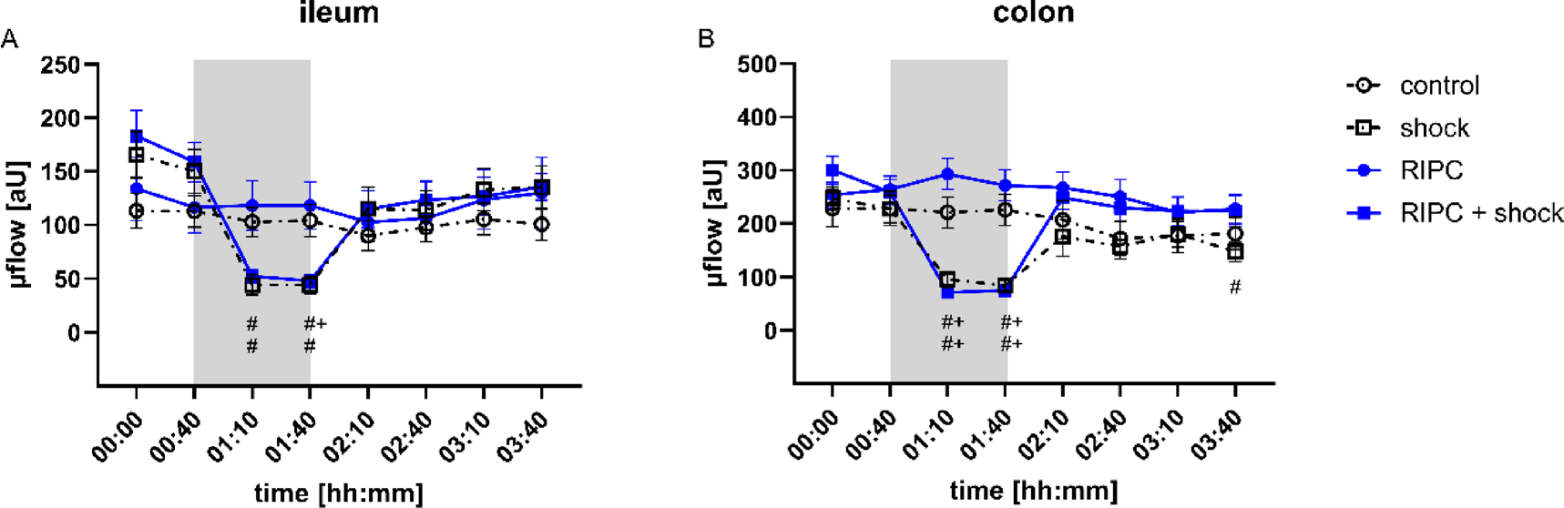
Laser Doppler flowmetry. Time-related changes of ileal (**A**) and colonic (**B**) microvascular blood flow (µflow; [aU]) after control treatment, remote ischemic preconditioning (RIPC) and/or hemorrhagic shock with subsequent shed blood transfusion (shock). 1 h of hemorrhagic shock is marked grey. Data are presented as mean ± SEM for *n* = 12 Wistar-rats, *p* ≤ 0.05. *: control vs. RIPC or shock vs. shock + RIPC; #: vs. individual baseline; +: control vs. shock or RIPC vs. shock + RIPC.

Measurement of baseline µflow revealed a wide interindividual variation of microvascular blood flow without significant differences between experimental groups (Fig. 6). The induction of acute hemorrhagic shock led to a significant decrease of ileal and colonic µflow when compared to the individual baseline. In colonic tissue these changes were also significant when compared to the normovolemic control group. Shed blood transfusion immediately reestablished baseline µflow values in both sections of the gastrointestinal tract. RIPC did not reveal beneficial effects on intestinal µflow during hemorrhagic shock and after shed blood transfusion. Calculating relative changes of µflow did not reveal improved µflow in animals with physiological hemodynamic conditions or hemorrhagic shock (Fig. S2).

### Incident dark-field imaging

**Fig. 7:**
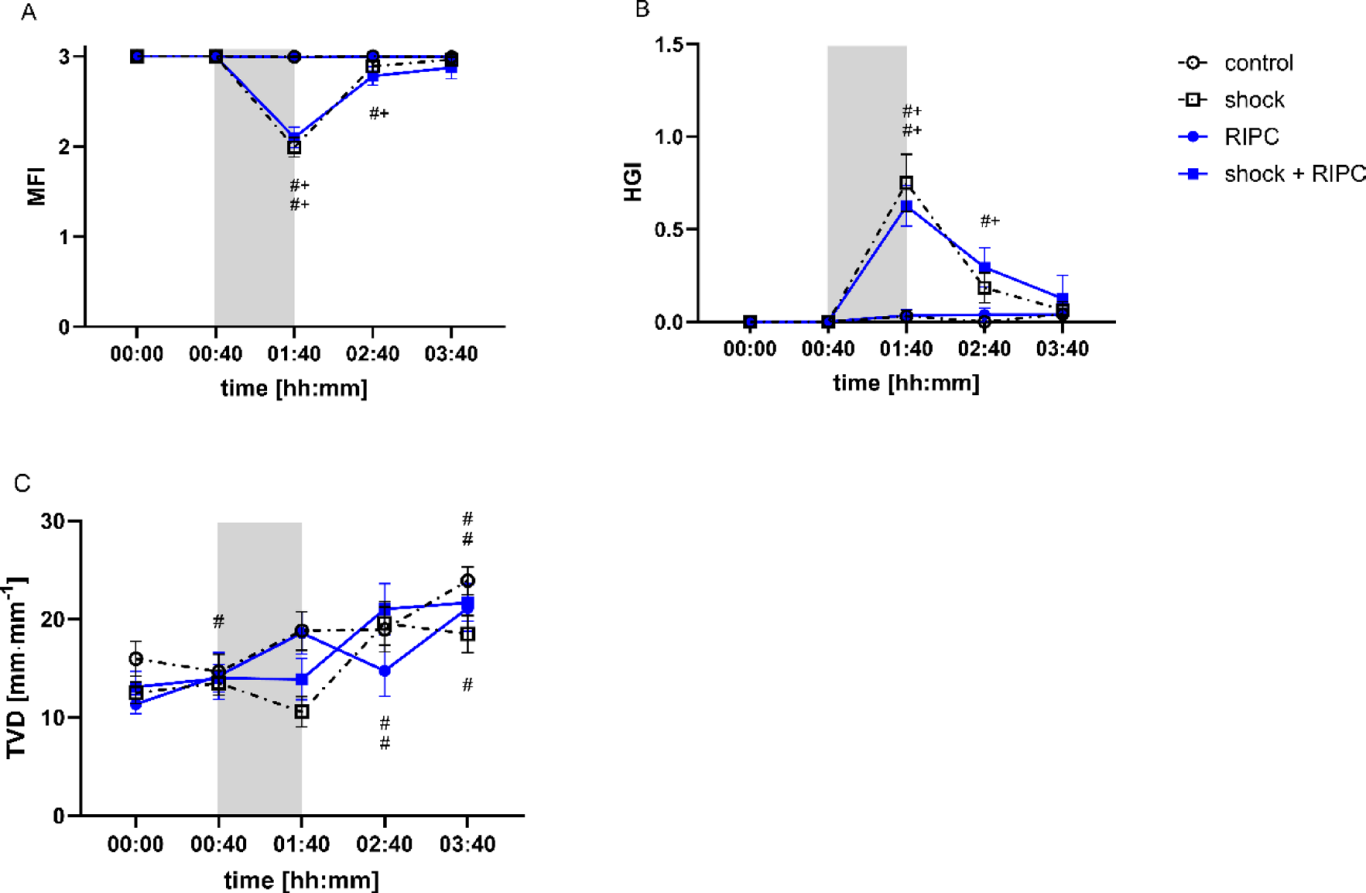
Incident dark-flied imaging. Time-related changes of Microvascular Flow Index (MFI; **A**], Heterogeneity Index (HGI; **B**) and Total Vessel Density (TVD; [mm·mm^− 1^]; **C**) after control treatment, remote ischemic preconditioning (RIPC) and/or hemorrhagic shock with subsequent shed blood transfusion (shock). 1 h of hemorrhagic shock is marked grey. Data are presented as mean ± SEM for *n* = 12 Wistar-rats, *p* ≤ 0.05. *: control vs. RIPC or shock vs. shock + RIPC; #: vs. individual baseline; +: control vs. shock or RIPC vs. shock + RIPC.

Microvascular flow index decreased in animals with hemorrhagic shock (Fig. 7A). These changes were significant when compared to the individual baseline and the normovolemic control group. Concordant to these findings, HGI increased after shock induction (Fig. 7B). RIPC neither improved colonic MFI nor HGI in animals with acute hemorrhage. Baseline values of MFI and HGI were reestablished by shed blood transfusion independent of RIPC. TVD increased in all experimental groups over time (Fig. 7C) without significant differences between the experimental groups. The induction of hemorrhagic shock did not reduce TVD.

### Mitochondrial respirometry

**Fig. 8:**
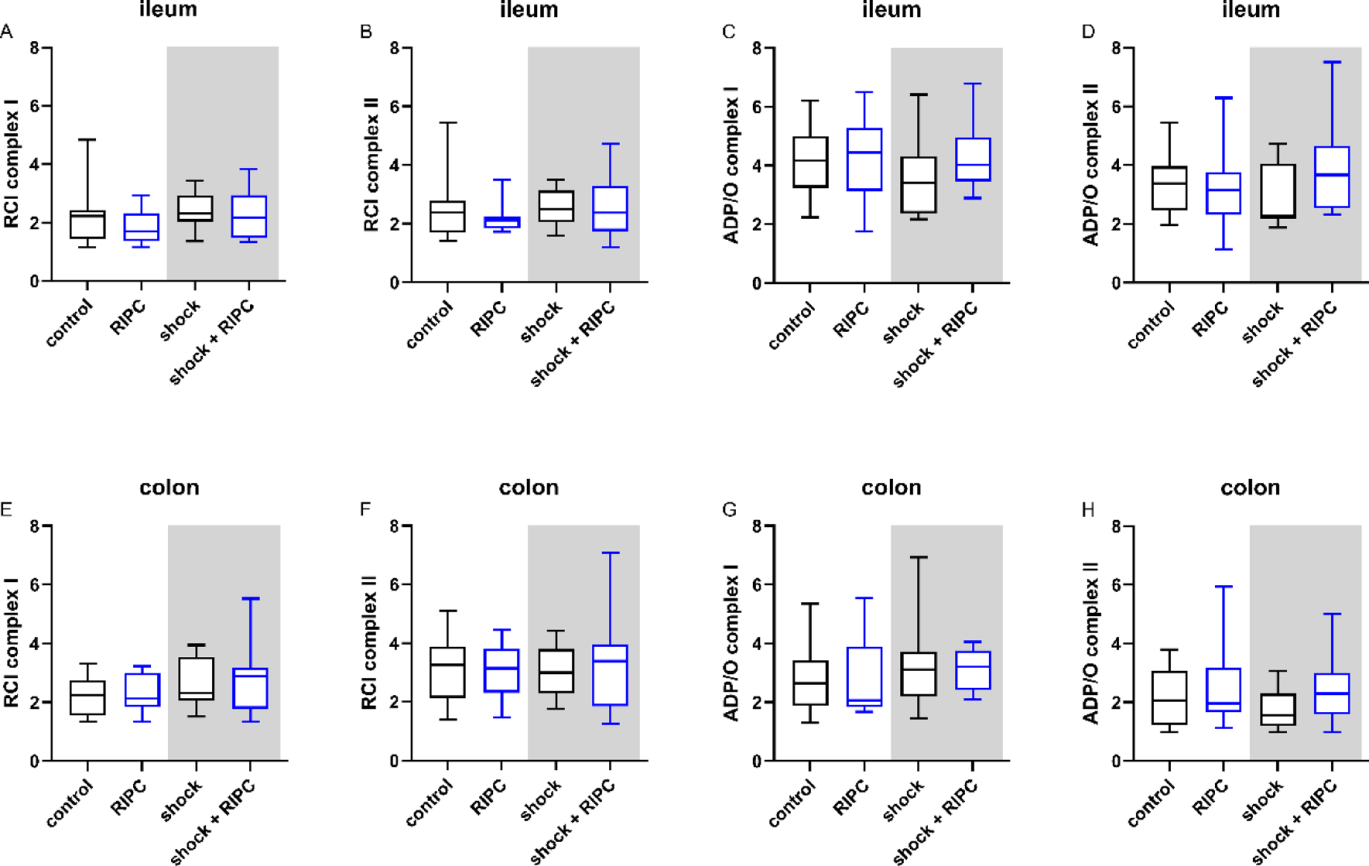
Mitochondrial respirometry. Respiratory Control Index (RCI) and ADP/O-ratio of ileal (**A**–**D**) and colonic (**E**–**H**) tissue samples measured ex-vivo via respirometry after selective complex I and complex II substrate supply. Data are presented for *n* = 12 as median and interquartile range. Whiskers indicate maximal and minimal values. Kruskal-Wallis testing and Dunn´s multiple comparisons did not reveal significant differences between the experimental groups.

Absolute respiration rates after hemorrhagic shock with subsequent shed blood transfusion did not reveal statistically significant differences between experimental groups (Fig. S3 and Fig. S4). In accordance, integrative variables of mitochondrial respiration were maintained in ileal and colonic tissue from animals that underwent hemorrhage reperfusion when measured ex-vivo by respirometry. RIPC did not reveal a significant effect on mitochondrial function under physiological conditions and after hemorrhagic shock with subsequent shed blood transfusion (Fig. 8). These results were independent of the stimulated complex of the respiratory chain and the investigated section of the gastrointestinal tract. Additional calculation of RCI* by dividing state 4 by state 3 did not change these findings (Fig. S5).

### MDA concentration

**Fig. 9:**
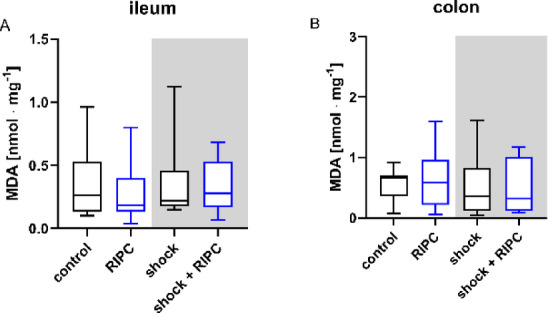
Malondialdehyde concentration. Ileal (**A**) and colonic (**B**) Malondialdehyde tissue concentration (MDA; [nmol·mg^− 1^]) was measured ex-vivo from tissue homogenates. Data are presented for a subset of measurements as median and interquartile range. Whiskers indicate maximal and minimal values. Kruskal-Wallis testing and Dunn´s multiple comparisons did not reveal significant differences between the experimental groups.

The MDA tissue concentration of the ileum and colon did not reveal altered lipidperoxidation after RIPC-treatment or hemorrhage retransfusion or the combination of both (Fig. 9).

### Intestinal tissue integrity

**Fig. 10:**
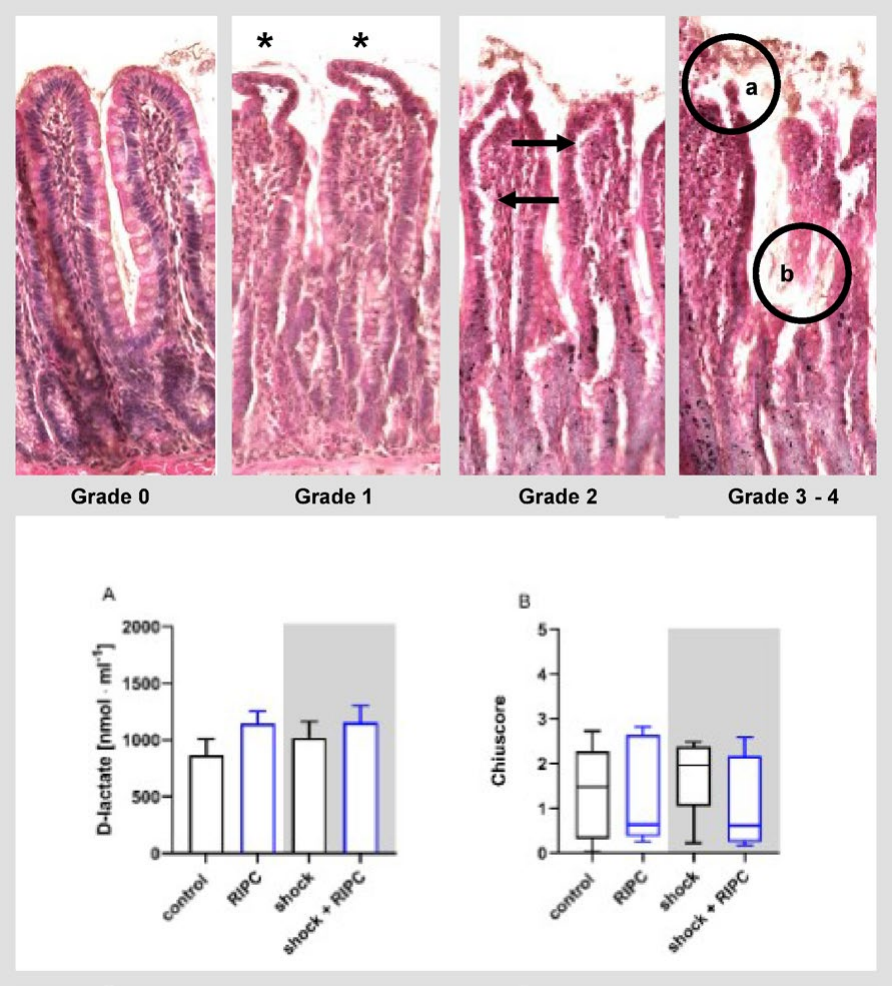
Intestinal tissue integrity. Intestinal tissue integrity was assessed by plasmatic D-lactate concentration (**A**; [nmol·ml^− 1^]) and histological scoring (**B**) according to Chiu et al.^35^. Animals received remote ischemic preconditioning (RIPC) and/or hemorrhagic shock with subsequent shed blood transfusion (shock). Shock groups are indicated by grey boxes. Control animals did not receive RIPC or hemorrhagic shock. Data are presented as mean + SEM for plasmatic D-lactate concentration and as median with interquartile range for histological scoring. Whiskers indicate maximal and minimal values. Statistical analysis did not reveal significant differences between the experimental groups. The upper row of images are giving an impression of the most present histological injury patterns from maintained tissue integrity (grade 0, left image) to progredient intestinal injury (grade 3–4, right image). Asterisks = Grunhagen space (Grade 1); Arrows = subepithelial lifting (Grade 2); Circle = epithelial loss with denuded apex (a; Grade 3) or villus (b; Grade 4).

Plasmatic D-lactate concentration did not differ between experimental groups (Fig. 10A). Evaluation of ileal tissue integrity using the Chiu scoring system revealed wide interindividual variability of histological tissue injury (Fig. 10B). Neither the induction of hemorrhagic shock with subsequent transfusion, nor RIPC or a combination of both had an effect on histological tissue integrity.

## Discussion

This study was conceptualized to investigate the effect of RIPC on microvascular oxygenation, regional microcirculation, and mitochondrial respiration of the intestine in a combined model of hemorrhagic shock with subsequent shed blood transfusion. We defined intestinal µHbO_2_ as the primary endpoint of this study since the detected light signal mainly indicates postcapillary oxygenation and therefore local oxygen reserve. RIPC significantly improved ileal µHbO_2_ during early hemorrhagic shock. This effect was not measurable in colonic tissue and was independent of regional microcirculation and mitochondrial respiration. Thus, the hypothesis, that RIPC exerts beneficial effects on intestinal µHbO_2_ after shed blood transfusion, could not be confirmed.

We used an established model of fixed-pressure hemorrhage that was reported to induce intestinal hypoxia with functional barrier loss^11^ and combined it with subsequent shed blood transfusion to investigate the effect of RIPC on microvascular oxygenation, regional microcirculation, and mitochondrial respiration in the context of blood reperfusion. According to the experimental protocol mean arterial blood pressure significantly declined by acute bleeding as verified by invasive blood pressure measurement. As reported in previous studies, macrocirculatory depression after arterial blood withdrawal was accompanied by a decrease of gastrointestinal µHbO_2_ as a measure of regional tissue hypoxia and a reduction of intestinal µflow and colonic MFI as variables of microvascular red blood cell perfusion^11^. TVD that wasderived from the automated analysis of IDF-imaging did not decrease during hemorrhagic shock. This may be due to the use of an analysis software that is still under development and lacks of sufficient validation. Recently, Müller-Graf et al. demonstrated that the automated analysis of the microcirculation using the manufactures software shows insufficient correlation with a manual evaluation^36^. This raises concerns that results gained from the provided automated microcirculatory assessments might be a subject to inaccuracies. In contrast to results from the automated microcirculatory assessment, MFI and HGI that were derived from manual assessment, mirrored hemorrhagic shock on a microvascular level. Severe tissue hypoxia during hemorrhagic shock led to systemic anaerobic metabolism as generally indicated by increased L-lactate values in animals with hemorrhagic shock. However, L-lactate concentration only increased with a delay and indicated systemic tissue hypoxia after shed blood transfusion and metabolic wash out of the microcirculation. This supports the need for opportunities to detect microcirculatory dysfunction and tissue hypoxia during shock in real time^37^. In contrast to arterial L-lactate concentration, µHbO_2_ and markers of red blood cell perfusion decreased immediately after the onset of acute hemorrhage and could therefore be more suitable to detect tissue hypoxia and microvascular hypoperfusion during shock. Further, µHbO_2_ and markers of red blood cell perfusion increased immediately after shed blood transfusion, and could also help to guide hemodynamic resuscitation as described in other experimental models of hemorrhagic shock^2^.

Unexpectedly, mean arterial blood pressure did not reach blood pressure values of the normovolemic control group after shed blood transfusion. Since citrate-phosphate-dextrose solution was used to store shed blood, this could be a result of negative inotropy as a consequence of calcium scavenging by citrate. However, arterial calcium concentration was unaltered in all experimental groups over time and did not reveal hypocalcemia in shock groups. Incomplete restoration of systemic hemodynamic variables can also be a result of either vasoplegia or persistent hypovolemia. Although increased formation of acid valences during hemorrhagic shock can potentially lead to vasoplegia, persistent hypovolemia might rather occur because of blood clot retraction by the transfusion filter. Although a reduced arterial hemoglobin concentration after shed blood transfusion would support this hypothesis, reduced hemoglobin values in the end of the experiment could also be the result of a compensatory fluid shift from the intercellular space into the luminal compartment of microvessels. However, the transfusion protocol used in this study is closely adapted to clinical transfusion triggers since final hemoglobin concentration in combination with the corresponding L-lactate values would not justify any further blood transfusion in human patients either^3^. In contrast to systemic hemodynamic variables, it has to be emphasized that intestinal µHbO_2_ and indices of microvascular blood flow reached baseline values after shed blood transfusion without differences to the respective normovolemic controls. This might indicate complete microvascular resuscitation after shed blood transfusion despite altered macrocirculatory variables. In fact, the goals of red blood cell transfusion are currently content of multiple debates with the trend towards restrictive transfusion goals^3^. This study extends the body of literature that absolute hemoglobin concentration and macrohemodynamic variables do not always reflect the status of microvascular blood flow and the need for further red blood cell transfusion^38^. Direct assessment of the microcirculation could help to redefine transfusion thresholds based on pathophysiological principles and meet the individual needs of tissue oxygenation demand more accurately^39^.

RIPC did not reveal systemic effects on hemodynamic variables, but improved ileal µHbO_2_ in the early course of hemorrhagic shock. This supports the hypothesis, that tissue oxygenation and regional microcirculation can be improved apart from systemic hemodynamic variables. In accordance, we reported in previous studies, that local treatment of the gastrointestinal tract using topical application of carbon dioxide^18^, nitroglycerine^40^, and carbachol^20^ exert beneficial effects on microvascular variables independent of systemic hemodynamic variables. As discussed in the introduction section, strategies to improve tissue oxygenation in patients with acute blood loss are especially needed for the early period of shock. RIPC exerted its main effect on ileal µHbO_2_ immediately after the onset of hemorrhagic shock, making the concept of RIPC suitable for clinical translation. However, RIPC procedure was performed prior to hemorrhagic shock in adaption to its initial description in the context of myocardial infarction^8^. Acute bleeding in acute and emergency medicine is mostly an unexpected and unpredictable event that does not allow the attending physician to perform a pretreatment to prevent tissue injury. In contrast, acute bleeding should be anticipated in patients undergoing major surgery that could profit from alternative strategies to ameliorate perioperative tissue injury by hemorrhagic shock. Santos et al. reported, that not only ischemic preconditioning but also ischemic postconditioning reduces intestinal tissue injury in a model of mesenteric ischemia-reperfusion injury^41^. If these results are transferable to the concepts of remote ischemic preconditioning and acute hemorrhage has to be clarified in further studies. In addition, the effect of RIPC on µHbO_2_ was limited in a regional specific manner to the ileal section of the gastrointestinal tract and in a time dependent manner to the first 30 min of hemorrhagic shock. If these changes can be attributed to the maintenance of global homeostasis in patients suffering from hemorrhagic shock remains uncertain.

However, it has to be clarified why RIPC revealed beneficial effects on ileal µHbO_2_ during hemorrhagic shock in the recent study but not in the previous one using animals with isolated hemorrhagic shock^11^. In both studies the targeted mean arterial blood pressure of hemorrhagic shock was set to 40 ± 5 mmHg. Invasive blood pressure measurement ensured that the targeted mean arterial blood pressure during hemorrhagic shock was reached accurately by intermittent blood withdrawal. However, baseline values of mean arterial blood pressure were higher in the study with isolated hemorrhagic shock than in the recent study leading to a higher relative blood pressure depression. A more accentuated relative depression of systemic hemodynamic variables could prostrate microcirculatory and cellular compensatory mechanisms and abolish beneficial effects of RIPC during hemorrhagic shock. Various confounders that interfere with the RIPC-related effect as the animals´ age and sex^42^, the used anesthetic^43^ and analgetic substances^44^ and the metabolic state^45^ have been identified in the past. It is possible that the surrounding hemodynamic conditions have to be added to this list. However, the known confounders of RIPC-related effects were excluded by our experimental protocol and does not explain the differences between both studies. Since this study was not designed to investigate the impact of baseline hemodynamic variables on RIPC derived intestinal tissue protection, further studies should investigate if RIPC-related beneficial effects on ileal microvascular oxygenation depend on baseline hemodynamic conditions. In the presented model of standardized and controlled fixed-pressure hemorrhage RIPC improved ileal microvascular oxygenation which could indicate increased reliance of the ileum against tissue hypoxia. Further investigations should clarify the impact of absolute and relative shock depth on RIPC induced effects. In a landmark paper on microcirculatory disorders during experimental hemorrhagic shock in sheep Dubin et al. reported similar trends across the results of sublingual microcirculatory assessment and serosal and mucosal microcirculatory measurements of the ileum^25^. A consecutive study targeting subsequent shed blood transfusion supported the existence of microcirculatory coherence during hemorrhagic shock and shed blood transfusion across different tissue compartments and measurement sites^2^. Using a second channel of white-light spectrophotometry, we assessed colonic µHbO_2_ in the recent experiments. In contrast to ileal µHbO_2_, colonic µHbO_2_ remained unchanged after RIPC-treatment in animals with hemorrhagic shock. These findings extend the recent body of literature gained by our research group, that different sections of the gastrointestinal tract might have a variable susceptibility to hypoxic events and therapeutic interventions that aim to reverse regional derangements^18–20,46^. Therefore, we cannot conclude that the findings derived from ileal microcirculatory assessment are fully transferable to other sites of the gastrointestinal tract.

Surprisingly, improved ileal µHbO_2_ values after RIPC were neither a result of improved microcirculation nor of altered mitochondrial respiration in the recent study. It can be assumed that RIPC led to a temporary reduction of cellular oxygen demand during hemorrhagic shock without affecting terminal energy metabolism on the mitochondrial level. Similar phenomena have already been described in the context of myocardial infarction and are referred to as hibernating myocardium^47^. However, one major limitation of assessing mitochondrial function by using respirometry in models of restricted oxygen delivery is, that respirometry had been performed ex-vivo after combined shock and shed blood transfusion and under normoxic conditions. Respirometry can therefore only indicate long-term effects of therapeutic interventions on mitochondrial respiration. This might explain unchanged ileal mitochondrial function after RIPC-treatment as well as preserved mitochondrial function in animals with isolated shock^11^ and combined hemorrhage transfusion. However, one can conclude that mitochondria reveal an extended reliance against temporary hypoxic challenges such as hemorrhagic shock^48^.

In last instance, therapeutic intervention targeting systemic hemodynamic variables, microvascular oxygenation, regional microcirculation and mitochondrial respiration are intended to beware tissue from progredient cell death and tissue injury. Therefore, we used histological staining and plasmatic injury markers to evaluate intestinal damage. Histological evaluation failed to mirror hemorrhagic shock possibly due to time related effects^11^. In fact, most of the studies harvest tissue samples 24 h after the onset of shock^15,49^. Further, experimental groups with hemorrhagic shock and shed blood transfusion did not reveal increased D-lactate concentration. In contrast, D-lactate significantly increased in animals with isolated hemorrhagic shock in the previous study^11^. Since both experimental protocols were identical except of the shed blood transfusion, unchanged D-lactate concentration in shock groups could be a result of dilution due to blood transfusion. Szalay et al. investigated time-related changes of plasmatic D-lactate concentration in a model of hemorrhagic shock and shed blood transfusion and reported, that shed blood transfusion leads to an immediate decrease of plasmatic D-lactate concentration^50^.

## Limitations

Despite the use of a well-established, standardized model of experimental hemorrhagic shock, this study is subject to some limitations. It should be mentioned that the experiments were conducted exclusively in animals of the same sex to reduce the number of animals required to elucidate statistically significant effects in accordance with the 3R initiative. We used male animals for three reasons. First, male animals are less subject to hormonal fluctuations than female animals. This further reduces the effect of hormonal influences as confounding factors compared to female conspecifics. Second, men have an increased risk of severe trauma and thus also of trauma-associated bleeding^51^. Male animals therefore better reflect the majority of the clinical patient collective than female animals. Thirdly, and perhaps most importantly, Heinen et al. were able to show that the transfer of plasma from male healthy volunteers that obtained RIPC, but not the transfer of plasma from female volunteers, induces tissue protection in a model of myocardial infarction in isolated rat hearts^42^. The aforementioned publication also showed that only young subjects are capable of producing factors that transduce tissue protection by RIPC. For this reason, only young animals were used in the current study. However, Crispens et al. could recently demonstrate, that the animals´ sex and age could be relevant to clinically significant outcome measures in experimental research especially when modelling critically illness^52^.

The shock model used in this study also has some limitations that have to be exposed. In contrast to hemorrhage models with a predetermined volume of blood withdrawal, the shed blood volume in fixed pressure hemorrhage models can vary as a result of endogenous compensatory mechanisms. However, since both systemic perfusion pressure and shed blood volume might have a direct effect on microvascular blood flow, one of these two variables has to be defined as the independent variable. In this study we used a fixed-pressure hemorrhagic shock model without registering and reporting the individual shed blood volumes. Since experimental hemorrhagic shock was exclusively guided by mean arterial blood pressure, shed blood volumes might differ between the experimental shock groups. Unfortunately, additional indicators of hypovolemia and volume responsiveness such as pulse pressure variation, systolic pressure variation or central venous pressure are only merely validated for the here used animal model and therefore were not calculated in the here used study. However, in most situations with severe bleeding it is unknown how much blood the patient has lost. Therefore, the attending physician can only assess systemic perfusion pressure to estimate the physiological relevance of the ongoing bleeding event^53^. By choosing a fixed pressure hemorrhage model, we aimed to mimic this situation as closely as possible.

Further, neither the induction of hemorrhagic shock nor the transfusion of shed blood was performed with a predetermined fixed rate protocol. A different bleeding time could lead to a variation of compensatory fluid mobilization from the extravascular to the intravascular space and subsequently to a variable increase of the circulating blood volume during hemorrhagic shock. However, this is an effect of hemodilution and will not necessarily increase microvascular red blood cell perfusion and regional tissue oxygenation. In the case of blood transfusion, there are no evidence-based protocols for emergency blood transfusion either in rats or in humans. Since extensive blood transfusion in trauma patients was related to endothelial dysfunction and glycocalyx shedding^54^, we performed a gradual blood transfusion considering systemic circulatory variables to avoid therapeutic volume overload.

Finally, we would like to point out that RIPC has been investigated as a potential intervention for preventing tissue damage, organ damage and hemorrhage-associated mortality, but that microcirculatory and mitochondrial changes as the endpoints of our study have essentially only been considered as precursors to these adverse events.

## Conclusion

Despite these limitations, which are caused by the chosen experimental design, the following statements can be made based on the data presented. Markers of microvascular red blood cell perfusion and local microvascular oxygenation indicate regional oxygen depletion of tissues at risk, even before the manifestation of irreversible tissue damage is apparent. RIPC might improve intestinal integrity by increasing local oxygen reserve. This finding was neither linked to an improved microcirculation nor to enhanced mitochondrial respiration. Whether these findings are also associated with a reduction of multiple organ failure requires further investigations. The effect of RIPC on intestinal oxygen reserve seems to depend not exclusively on the absolute depth of hemorrhagic shock, but on the relative changes of systemic hemodynamic variables and the investigated section of the gastrointestinal tract. Therefore, a deeper understanding of the molecular mechanisms of RIPC is of paramount importance for future therapeutic applications.

## Supplementary Information

Below is the link to the electronic supplementary material.

Supplementary Material 1

## Data Availability

The datasets used and analyzed during the current study are available from the corresponding author on reasonable request.
