# Supplementary material for: Remote ischemic preconditioning improves ileal microvascular oxygenation during rodent hemorrhagic shock without improving variables of microcirculation and mitochondrial respiration
Source: Sci Rep. 2025 Dec 4;15:43230. doi: 10.1038/s41598-025-29245-2 (PMC12680664; doi:10.1038/s41598-025-29245-2)
Supplement: Supplementary file 1 — Supplementary Material 1 [file 41598_2025_29245_MOESM1_ESM.docx]

**Supplemental data**


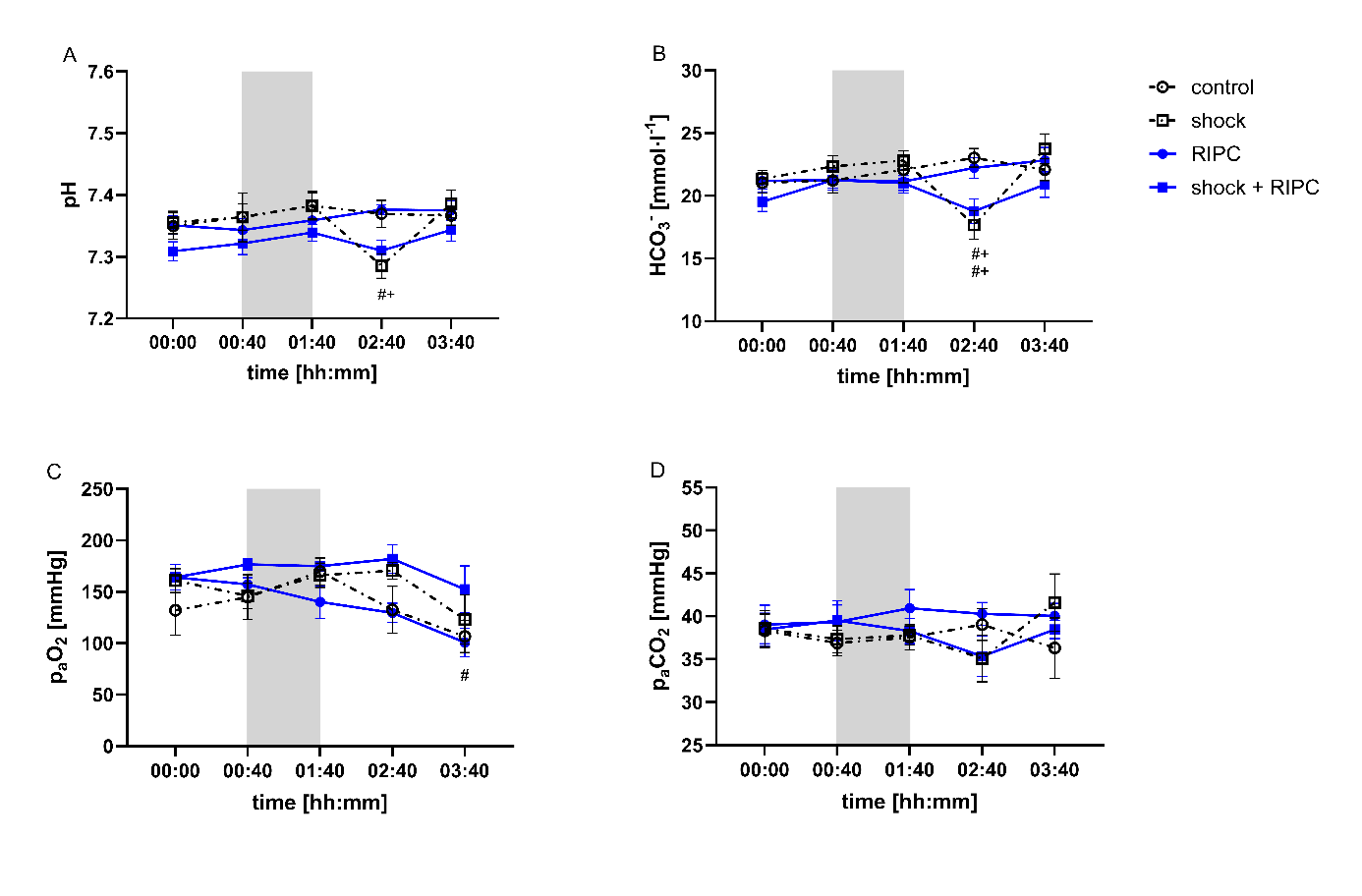


**Fig. S1: Acid-base status and arterial blood gas analysis**. Time-related changes of pH-values (A), arterial bicarbonate concentration (HCO_3_^-^; [mmol·l^-1^]; B), arterial oxygen partial pressure (p_a_O_2_; [mmHg]; C) and arterial carbon dioxide partial pressure (p_a_CO_2_; [mmHg]; D) after control treatment, remote ischemic preconditioning (RIPC) and/or hemorrhagic shock with subsequent shed blood transfusion (shock). 1 h of hemorrhagic shock is marked grey. Data are presented as mean ± SEM for n = 12 Wistar-rats p ≤ 0.05. #: vs. individual baseline; +: control vs. shock or RIPC vs. shock + RIPC.


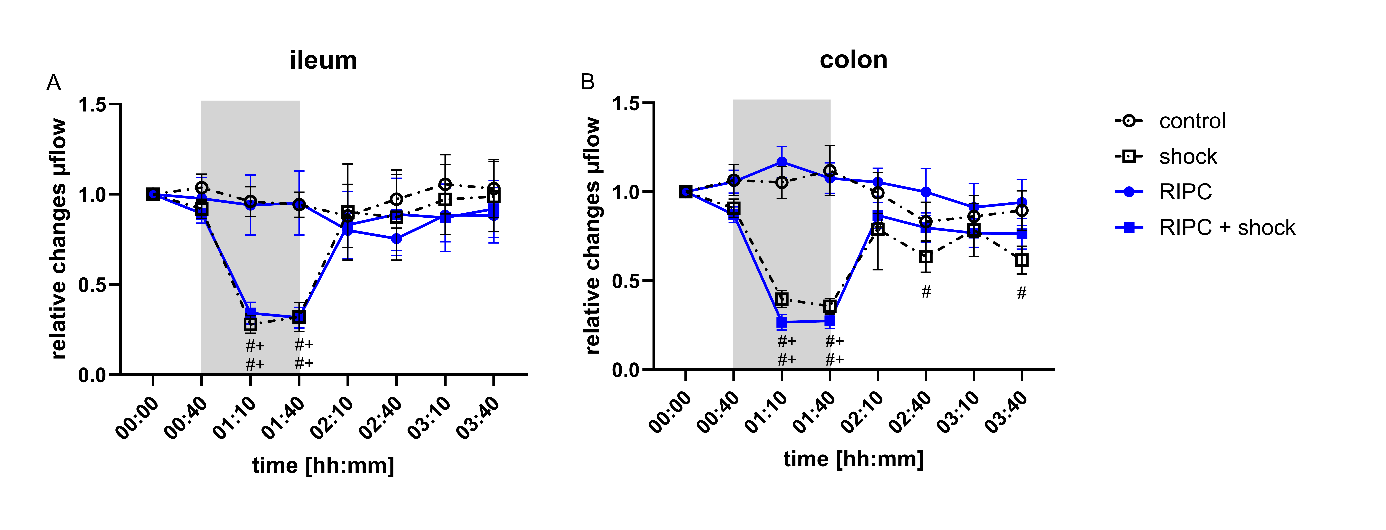


**Fig. S2: Relative changes of microvascular blood flow.** Time-related relative changes of ileal (A) and colonic (B) microvascular blood flow (µflow) after control treatment, remote ischemic preconditioning (RIPC) and/or hemorrhagic shock with subsequent shed blood transfusion (shock). 1 h of hemorrhagic shock is marked grey. Data are presented as mean ± SEM for n = 12 Wistar-rats, p ≤ 0.05. *: control vs. RIPC or shock vs. shock + RIPC; #: vs. individual baseline; +: control vs. shock or RIPC vs. shock + RIPC.


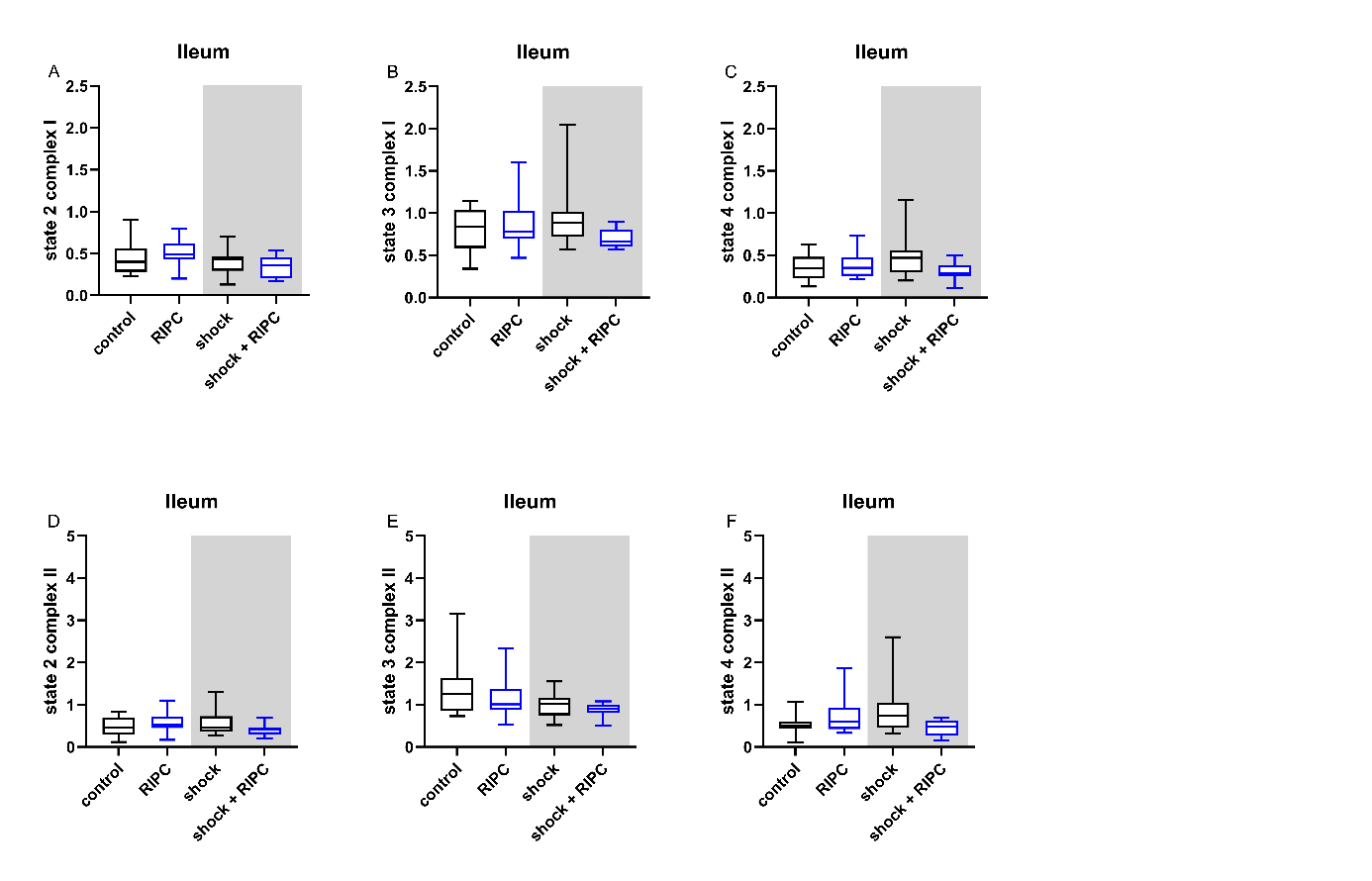


**Fig. S3: Respiratory states of the ileum.** Respiratory states of ileal tissue samples measured ex-vivo via respirometry after selective complex I (A, B, C) and complex II (D, E, F) substrate supply. Data are presented for n = 12 as median and interquartile range. Whiskers indicate maximal and minimal values. Kruskal-Wallis testing and Dunn´s multiple comparisons did not reveal significant differences between the experimental groups.


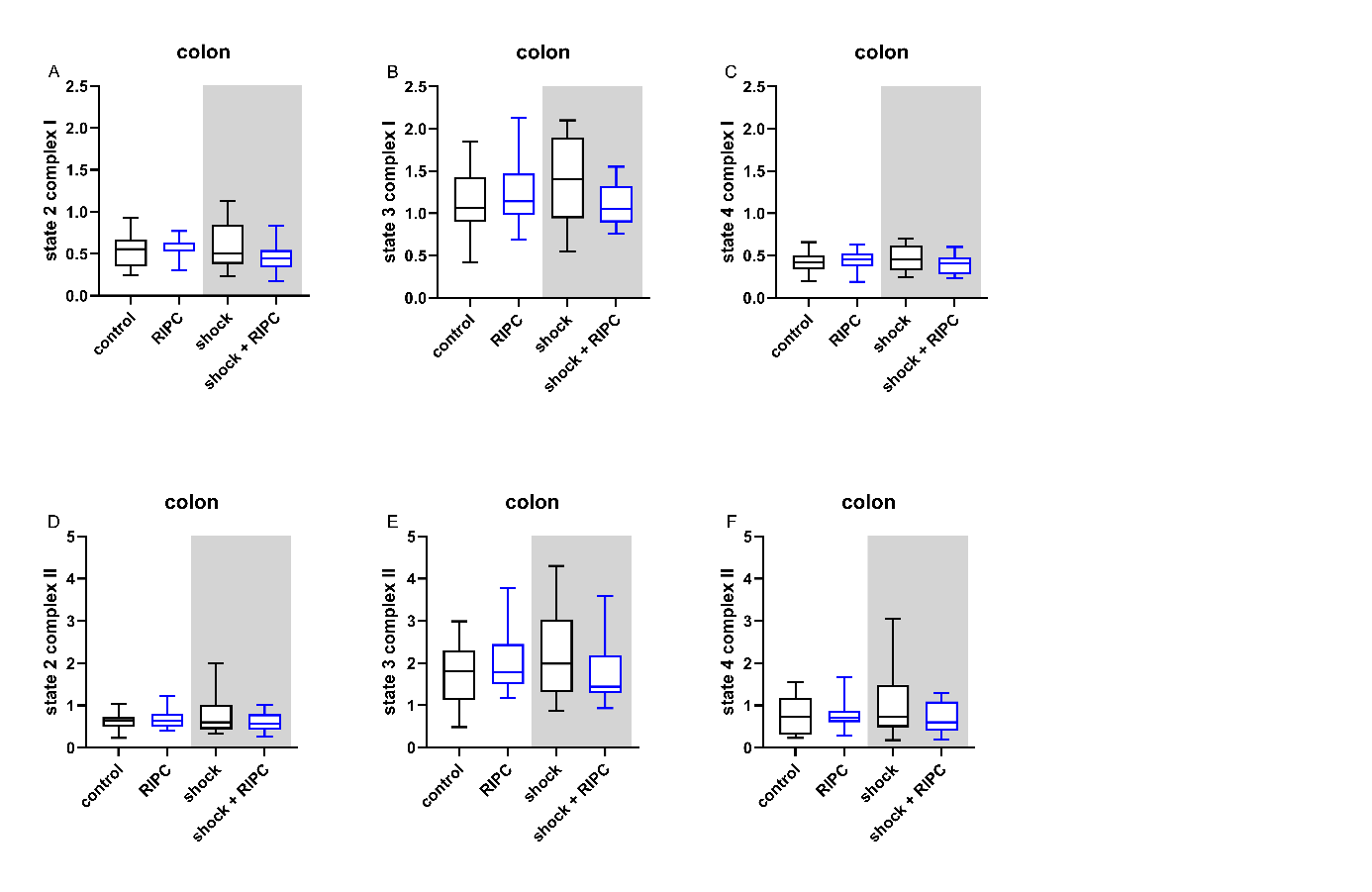


**Fig. S4: Respiratory states of the colon**. Respiratory states of colonic tissue samples measured ex-vivo via respirometry after selective complex I (A, B, C) and complex II (D, E, F) substrate supply. Data are presented for n = 12 as median and interquartile range. Whiskers indicate maximal and minimal values. Kruskal-Wallis testing and Dunn´s multiple comparisons did not reveal significant differences between the experimental groups.


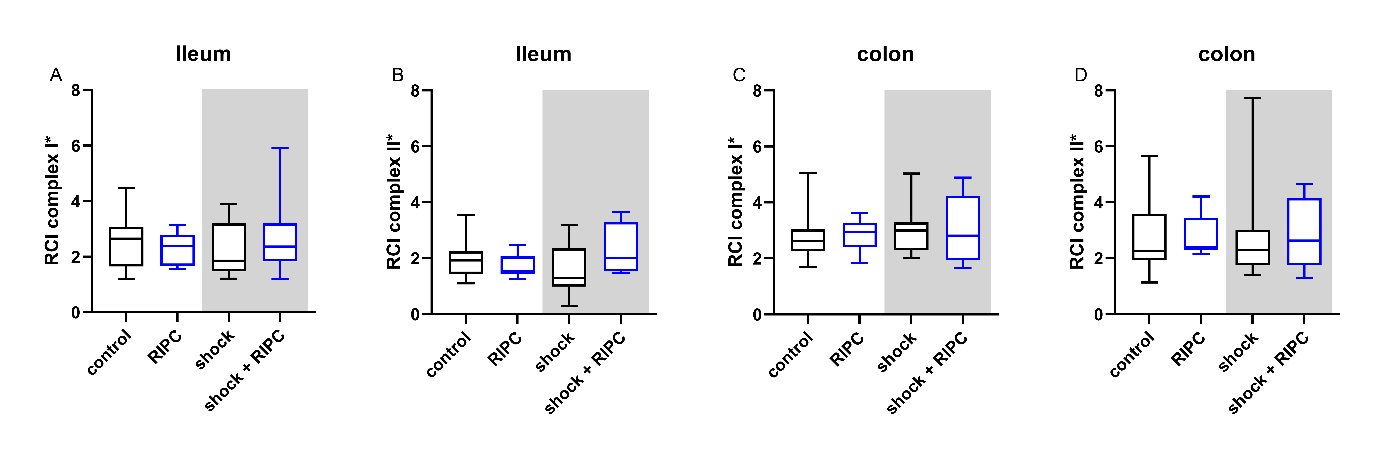


**Fig. S5: Modified respiratory control index.** Alternative calculation of the respiratory control index of ileal (A, B) and colonic (C, D) tissue samples measured ex-vivo via respirometry after selective complex I (A, C) and complex II (B, D) substrate supply. Data are presented for n = 12 as median and interquartile range. Whiskers indicate maximal and minimal values. Kruskal-Wallis testing and Dunn´s multiple comparisons did not reveal significant differences between the experimental groups.
